# What motivates GPs to train medical students in their own practice? A questionnaire survey on the motivation of medical practices to train students as an approach to acquire training practices

**DOI:** 10.3205/zma001633

**Published:** 2023-06-15

**Authors:** Louisa Daunert, Sven Schulz, Thomas Lehmann, Jutta Bleidorn, Inga Petruschke

**Affiliations:** 1 University Hospital Jena, Institute for General Medicine, Jena, Germany; 2 University Hospital Jena, Institute for Medical Statistics, Informatics and Data Science, Jena, Germany

**Keywords:** teaching motivation, outpatient training, general medicine, teaching practice, medical students

## Abstract

**Background:**

With the new licensing regulations for doctors coming into force, medical faculties are faced with the task of recruiting and qualifying more GP colleagues to train students in their practices.

**Objective:**

The aim of the study was to determine the motivation of GPs to train students in their own medical practices.

**Method:**

A cross-sectional survey of Thuringian GPs was conducted from April to May 2020. 21 items on motivation, incentives and barriers were recorded and examined using univariate and multivariate analyses.

**Results:**

The response rate was 35.8% (538/1,513). The GPs surveyed considered themselves motivated to train students in their medical practices. The motives can be described as predominantly intrinsic: the mutual exchange of knowledge, desire to share knowledge and to promote future doctors. Incentives included the opportunity to keep up to date with the latest knowledge, further training and contacts with colleagues. Barriers to teaching in the own medical practice were concerns about not being able to treat the same number of patients, a possible disruption of practice operations and lack of space. An analysis of the subgroups of GPs who were not yet teaching physicians indicated similar motives and barriers regarding training students in their practices, with a slightly lower overall motivation.

**Conclusion:**

The results describe the facets of the motivation of Thuringian GPs to train students and can be helpful for the expansion of a sustainable network of training GP practices. It is essential to address motives, to counter difficulties with individual solutions and to create targeted incentives.

## Zusammenfassung

**Hintergrund:**

Mit Inkrafttreten der neuen Approbationsordnung für Ärzte stehen die Medizinischen Fakultäten vor der Aufgabe, weitere hausärztliche Kolleg*innen für die Ausbildung Studierender in ihren Praxen zu gewinnen und zu qualifizieren.

**Zielsetzung:**

Ziel der Studie war die Erfassung der Motivation von Hausärzt*innen zur Ausbildung Studierender in der eigenen Praxis.

**Methodik:**

Von April bis Mai 2020 wurde eine Querschnittsbefragung von Thüringer Hausärzt*innen durchgeführt. 21 Items zu Motivation, Anreizen und Barrieren wurden erfasst und mittels univariater und multivariater Analysen untersucht.

**Ergebnisse:**

Der Rücklauf betrug 35,8% (538/1.513). Die befragten Hausärzt*innen schätzten sich motiviert ein, Studierende in ihrer Praxis auszubilden. Die Motive sind als überwiegend intrinsisch zu beschreiben: beidseitiger Wissensaustausch, Lust, Wissen zu teilen und den Nachwuchs zu fördern. Anreize waren die Chance, selbst auf dem neuesten Stand des Wissens zu bleiben, Fortbildungen und kollegiale Kontakte. Als hinderlich für Lehre in der eigenen Praxis zeigten sich die Sorge, weniger Patient*innen versorgen zu können, eine mögliche Störung des Praxisablaufs und fehlende Räumlichkeiten. Eine Subgruppenanalyse der Hausärzt*innen, die noch keine Lehrärzt*innen waren, nannten ähnliche Motive und Barrieren hinsichtlich der Ausbildung Studierender in ihrer Praxis bei einer insgesamt etwas geringeren Gesamtmotivation.

**Schlussfolgerung:**

Die Ergebnisse beschreiben die Facetten der Lehrmotivation unter Thüringer Hausärzt*innen und können beim Ausbau eines nachhaltigen, hausärztlichen Lehrpraxennetzwerks hilfreich sein. Motive sollten adressiert, Schwierigkeiten mit individuellen Lösungen begegnet und zielgerichtete Anreize geschaffen werden.

## 1. Introduction

More general medicine as part of the medical curriculum, more training in the outpatient sector – these recommendations of the Medical Studies Master Plan 2020 [1] are substantiated in the draft bill of the new Medical Licensing Regulations (Ärztlichen Approbationsordnung (ÄApprO)). This means that training GP practices will be included in medical education to a much greater extent than before [2]. In addition to a compulsory quarter as part of the practical year in outpatient statutory health care, an extension of the current two-week block internship in general medicine is expected. This means that the medical faculties are faced with the task of recruiting more training GP practices in the coming years and qualifying them to train students. Teaching physicians who train students in their practices or medical centres are usually contractually bound and receive varying levels of remuneration depending on the faculty.

### 1.1. Motivation

A prerequisite for assuming a teaching activity in addition to patient care is motivation. According to the model of “classical motivation psychology” by Rheinberg and Vollmeyer, the combination of person-related motives and a suitable situation leads to the emergence of “motivation” [3]. In addition, one can speak of situational incentives that act on a person or situation and result in an action [4]. Relevant in the study of motivation – in our survey of teaching motivation – is to capture both intrinsic (Why do I want to do something?) and extrinsic motives (What do I get out of doing something?) [5], [6].

Surveys in the USA and Australia revealed a predominantly high motivation to teach among GPs [7], [8]. The influence of intrinsic motives (passing on knowledge, responsibility, enjoyment of teaching) and extrinsic incentives (advanced training, CME points, access to literature, recognition, remuneration) [5] on teaching motivation were established [7], [8], [9].

Current studies within Germany in the GP setting also show a high motivation to train students [10], [11]. The desire to share knowledge and to promote future doctors were named as motives for teaching; the interest in further training and networks were identified as incentives [10], [12], [13].

### 1.2. Question

In Thuringia, around 220 GP colleagues are contractually bound to the Medical Faculty of the Friedrich Schiller University of Jena and train students in the block internship and in the elective trimester “general medicine” as part of the practical year. In addition, many GPs gain experience with students as part of the compulsory internship in primary care, which has been obligatory since 2013 ([https://www.bundesgesundheitsministerium.de/themen/gesundheitswesen/gesundheitsberufe/aerzte.html], accessed on: 10/08/2022) and for which a connection as an academic teaching practice is not a prerequisite. For the implementation of the upcoming licensing regulations, there is a need for further GP practices that are qualified as academic teaching practices. The aim of the survey was therefore to collect current and regional data and to create a scientific basis for concepts and recommendations for recruiting teaching physicians.

## 2. Methods

### 2.1. Questionnaire

The two-page questionnaire was based on the questionnaire used by Adarkwah et al. [12]. The motivating factors were divided into intrinsic motives (promotion of future doctors, desire to share knowledge, social responsibility [...]) and extrinsic incentives (opportunity for contacts and networks, succession, academic teaching practice as an upgrade [...]) [3], [5]. The items were agreed upon by the team of authors. After piloting with five GP colleagues who did not participate in the survey, three items were adapted. Eleven socio-demographic and practice-specific characteristics were recorded, as well as 21 items on the dimensions of motives, incentives and barriers. The answers were given via four-point Likert scales (concurrence with statements) and free text for three items.

The project was approved by the ethics committee of the University Hospital Jena on 23/04/2020 (Reg.-no.: 2020-1753-Bef.).

### 2.2. Data collection, processing and analysis

At the end of April 2020, the questionnaire and survey information were sent to all GPs, internists and medical practitioners (n=1,513) in Thuringia. No compensation for efforts was paid. 

The returned questionnaires were entered into an SPSS file (IBM SPSS Statistics 27) via a document scanner and checked manually. In this way, missing information could be added and duplicate submissions identified. For almost all items, the number of missing data was below 5%. One item was excluded from the analysis, because the wording of the question proved to be too imprecise. The non-responder survey was not analysed due to the low response rate.

For the univariate analyses, overall motivation was defined as the target variable with the question “How motivated would you rate yourself to train students in your medical practice?”. The influence variables consisted of socio-demographic information, as well as individual items of motives, incentives and barriers. For simplification, the ordinally scaled items were dichotomised [14]. The correlation between metric or ordinally scaled influence variables and overall motivation was tested univariately using a two-sided Mann-Whitney U test. The relationship between categorical variables and overall motivation was tested using Fisher's exact test or the chi-square test (for more than two values). The items significant in the univariate analysis were included as influence variables in a multivariate, binary logistic regression with overall motivation as the outcome variable. Using the item “Is your practice an academic teaching practice of the University of Jena?” (n=331/538), the data set was split and the subgroup of non-teaching physicians was analysed. A qualitative content analysis of the free texts was performed using the MAXQDA software (version 2018).

## 3. Results

538/1,513 GPs participated in the survey; the response rate was 35.8%. The respondents were predominantly female, on average 52 years old and mainly working as owners in single medical practices. A total of 72.7% reported experience with students; 38.2% were working in teaching practices, see table 1 (Tab. 1).

### 3.1. Descriptive analysis of the total sample

**Figure 1:**
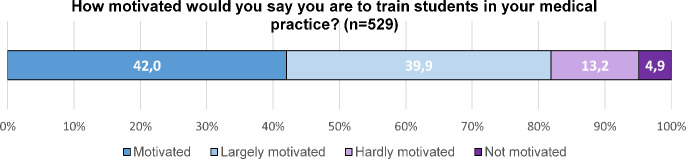
Motivation to train students in the own medical practice

The respondents indicated that they were highly motivated to train students in their own practice: The majority (81.9%) described themselves as motivated or motivated to a large extent, see figure 1 (Fig. 1).

There was a high level of agreement (applicable + rather applicable) with intrinsic motives: *Contribution to promoting future doctors* (87.6%), *knowledge exchange* (89.4%), *desire to share knowledge *(87.5%) and *social responsibility* (82.8%).

The four incentives with the highest agreement (applicable + rather applicable) were: Keeping *up to date with the latest knowle*dge (82.6%), the designation *academic teaching practice as an upgrade* (62.4%), the opportunity to participate in *advanced training* (58.3%) and the *opportunity for contacts* and networks (55.1%).

The three incentives of remuneration (46.1%), *access to literature via the university library* (45.5%) and *being perceived as more competent* (40.1%) received significantly less agreement.

Overall, possible barriers were affirmed less often than motives or incentives. The highest level of agreement was for *concerns about not being able to treat the same number of patients* (34.3%) and that students *could disrupt practice operations* (30.8%).

### 3.2. Multivariate analysis of factors influencing teaching motivation (total sample)

Respondents wanting to *share their knowledge* (OR: 18.2; p<0.001) and wanting to *contribute to the promotion of future doctors* (OR: 18.1; p<0.001) were most likely to be motivated to train students at their practice. Regarding* training as a social responsibility* (OR: 2.9; p=0.009) increased the likelihood, as did the desire to *stay abreast of the latest knowledge* (OR: 3.6; p<0.001), e.g., through *receiving training free of charge* (OR: 2.9; p<0.001) and *access to literature* (OR: 2.0; p=0.21), and the *opportunity to build contacts and networks* (OR: 2.5; p=0.002). If the practice had adequate facilities (space), respondents were more likely to report being motivated to train students in their own practice (OR: 8.1; p<0.001). 

Older respondents showed less motivation to train students in their own practice (OR: 0.95; p<0.001). Also *concern about disruption of practice operations* (OR: 0.4; p=0.013) and the *concern about not being able to treat the same number of patients* (OR: 0.4; p=0.003) reduced the likelihood of wanting to train students in the own medical practice.

### 3.3. Remuneration

The question *“What daily rate do you consider appropriate for training students in your practice?”* was answered by 57.1% of all respondents, with 29.6% not giving a specific answer but answering the question with “don’t know”. In free text, an average of €56/day (median €50/day) was indicated as reasonable, with a range of 0-€400/day.

### 3.4. Descriptive analysis of the subgroup of non-teaching physicians

**Figure 2:**
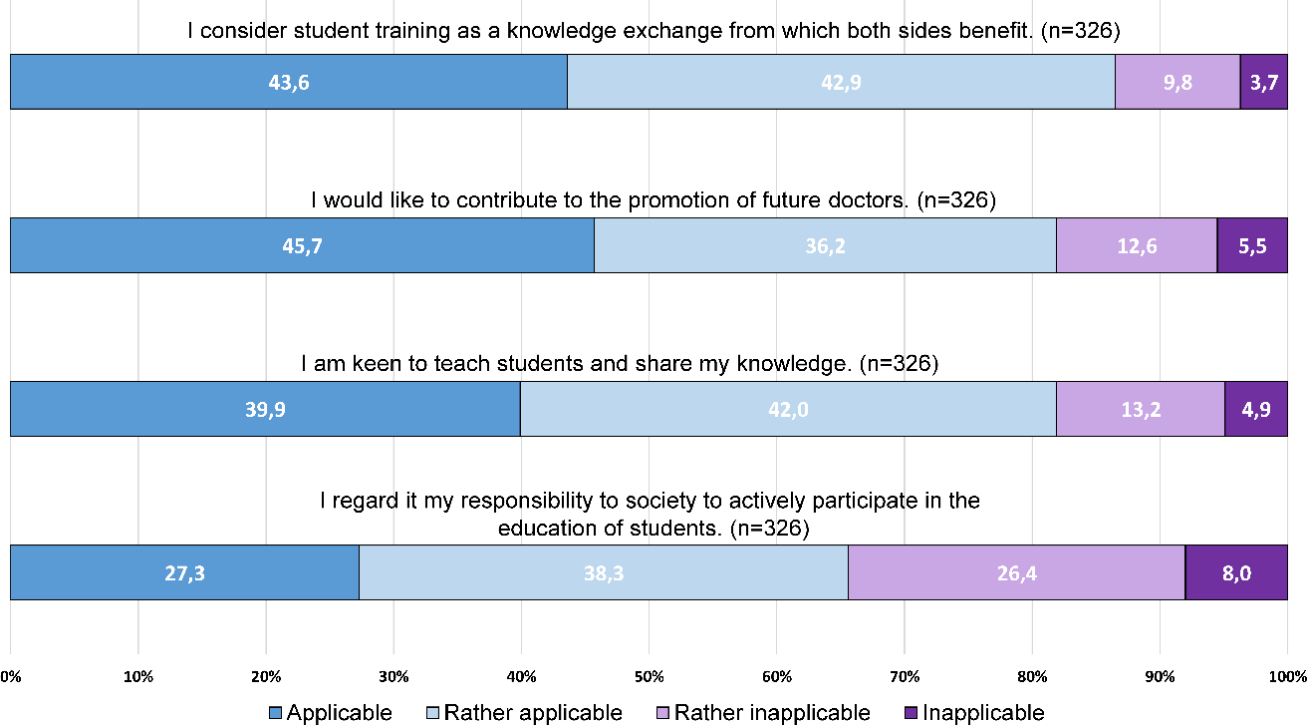
Concurrence with motives regarding student training in the own medical practice, subgroup of non-teaching physicians (n=331)

Of the respondents, 61.8% were not working in a teaching practice. These respondents indicated that they were also *motivated* (29.6%) or *largely motivated* (43.8%) to train students in their own practice (see figure 2 (Fig. 2)).

**Figure 3:**
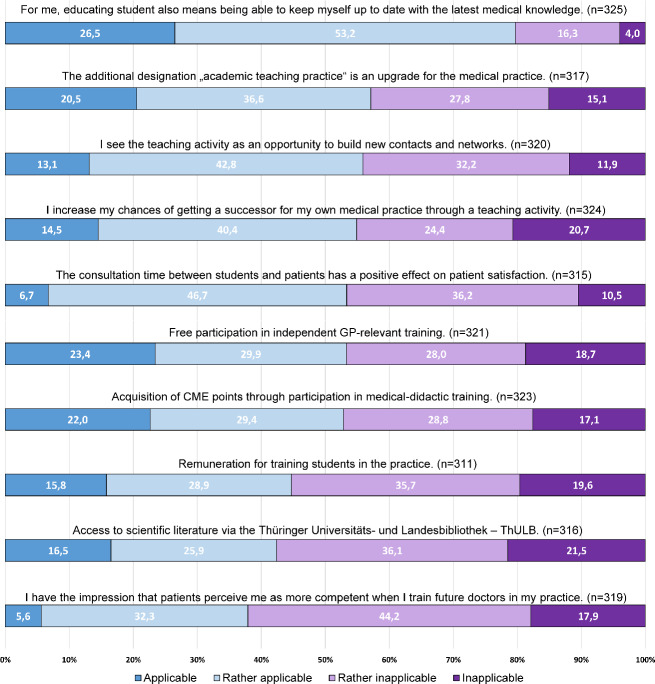
Concurrence with incentives regarding student training in the own medical practice, subgroup of non-teaching physicians (n=331)

For the motives, *knowledge exchange, contribution to promoting future do*ctors and the *desire to share knowledge*, the differences to the total sample were small, the agreement (applicable + rather applicable) was also >80%. *Social responsibility* as a motive for teaching showed an agreement rate in the subgroup, 65.6% vs. 73.0% in the total sample. The analysis of incentives showed no differences compared to the total sample (see figure 3 (Fig. 3)).

**Figure 4:**
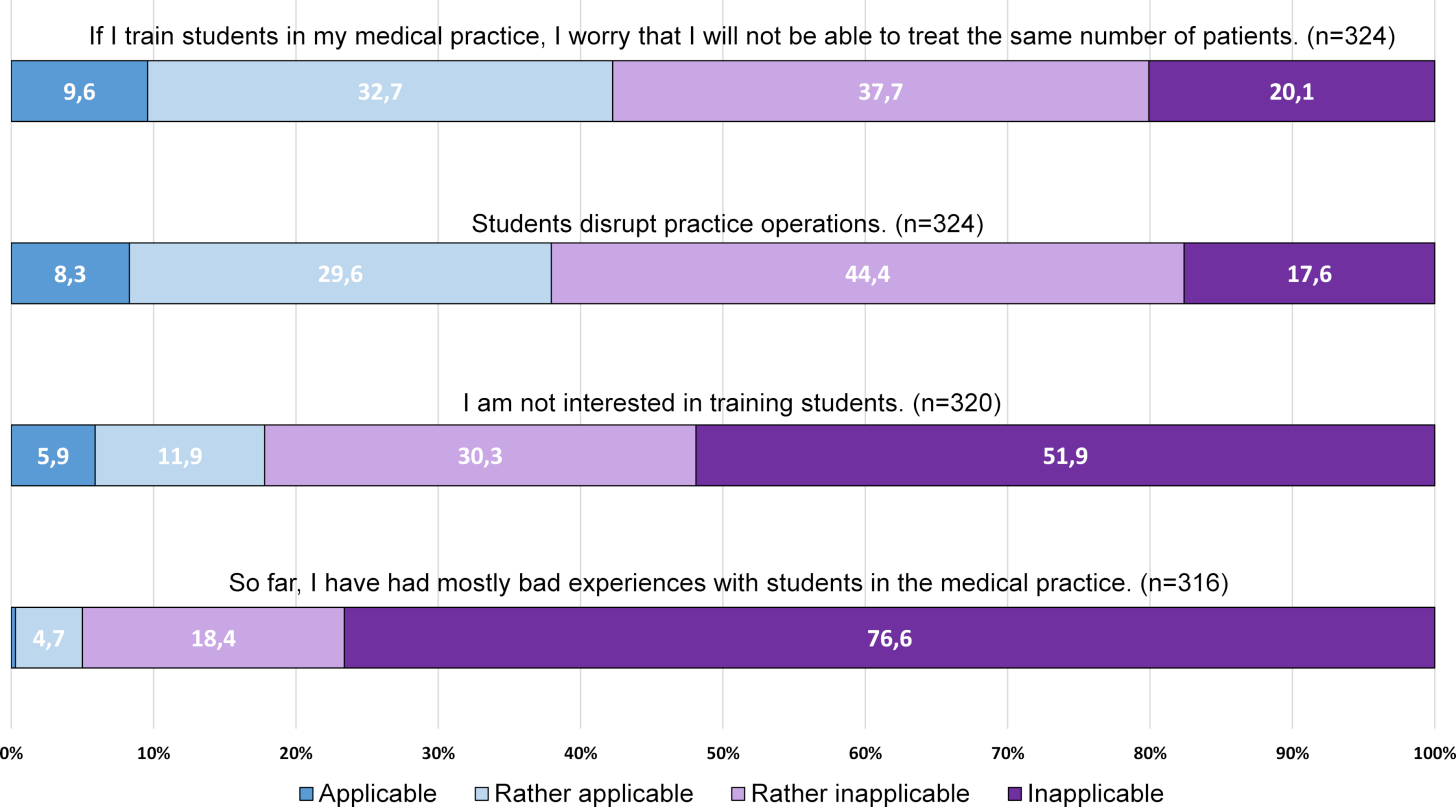
Concurrence with barriers regarding student training in the own medical practice, subgroup of non-teaching physicians (n=331)

The barriers were agreed with slightly more often in this subgroup, for example *concern about not being able to treat the same number of patients* was expressed more often (42.3% vs. 34.3% total sample); as was concern of a *suspected disruption of practice operations* (37.9% vs. 30.8% total sample), (see figure 4 (Fig. 4)).

### 3.5. Multivariate analysis of factors influencing teaching motivation (non-teaching physicians)

Respondents who had not previously worked as teaching physicians were more motivated to teach if they regarded training of students in their own practice as a* contribution* *to promoting future doctors* and if they wanted to share their knowledge. These were also the main motives of the total sample, but the OR for *contributing to the promotion of future doctors* was almost twice as high (OR: 34.8 vs. OR: 18.1). Furthermore, the incentives *opportunity for contacts and networks, student consultation time increases patient satisfaction* and *keeping up to date with the latest knowledge* increased the likelihood of wanting to train students in the own practice.

Higher age reduced the likelihood that respondents reported being motivated to train students (see table 2 (Tab. 2)).

## 4. Discussion

The purpose of this survey was to determine the motivation of Thuringian GPs to train medical students in their own practice, in order to expand the cooperation between GPs and the faculty on the basis of knowledge of the motives, incentives and barriers.

The 538 participating GPs roughly correspond to the average of Thuringian GPs in terms of age and gender, so it can be assumed that the results are valid for Thuringia as a whole [15].

### 4.1. Motivation

Overall, more than 80% of the respondents are highly motivated to participate in the training of students. This confirms the results of other surveys among GPs in Germany [10], [12], [13]. The high motivation to teach in our study population is also reflected in the fact that 73% of the respondents already had experience with students and 38% work in teaching practices. In a recent survey of GPs in Saxony, this proportion was significantly lower at 19% [10].

The most motivating factors in our survey (*contribution to promoting future doctors, benefiting from the exchange of knowledge* and* the desire to share knowledge*) can be seen as intrinsic and outweigh extrinsic incentives such as *remuneration, further training*, etc. This is also known from other studies on the training of students in a GP practice [7], [8], [13], [16], [17], [18], [19]. Studies from the USA and Australia showed that GPs find the exchange with students as beneficial and informative [8], [9], [18]. While the students benefit from the clinical experience of the teaching physicians, some of whom have many years of experience, the latter regularly reflect on their knowledge and procedures through questions, discussions and professional exchange with the students.

The intrinsic motive to contribute to the next generation of doctors by training students also plays an important role. Many GPs want committed young doctors, not least for their own practice. A publication from 2018 emphasises that training students in one's own practice also goes hand in hand with getting to know the next generation and the opportunity to inspire them for one’s own job [20]. Since one third of the GPs currently working in Germany are older than 60 [21], the search for a successor takes on great importance.

### 4.2. Incentives

In addition to the desire to keep up to date with the latest knowledge by training students in the own practice, the offer of further training and access to literature proved to be relevant incentives. In other surveys, the desire for feedback and evaluation for one's own work [10] and access to clinical databases were also expressed [9].

Building contacts and better networks through training students in one's own practice is also an incentive, although less relevant for the respondents here overall. Nevertheless, face-to-face training (also on medical didactic topics), meetings of teaching physicians and personal feedback can give recognition and create a sense of belonging that strengthens intrinsic motivation.

Remuneration was not a relevant incentive in our survey. This does not seem surprising given the predominantly intrinsic motivation of the respondents and is in line with studies from the USA, Canada and Germany [7], [13], [16], [22], [23]. The fact that 30% of the respondents did not specify a daily rate that is appropriate for them could be an indication of uncertainty, as a comparative figure is lacking. However, as several papers point out that the increased (time) effort is compensated [10], [24], the challenge is to find a level that avoids the “corrupting effect” [25] (reward reduces intrinsic motivation) and still does justice to the increased effort.

### 4.3. Approaches to recruiting teaching practices

For the recruitment of further general medical teaching practices, the responses of the non-teaching physicians are particularly interesting. In this group, the proportion of GPs motivated to train students was 73.4%. 

In case of the intrinsic motives (*knowledge exchange, contribution to promoting future doctors* and *the desire to share knowledge*), the differences to the overall sample were small, with agreement exceeding 80% in each case. In the subgroup of non-teaching physicians, the motive of *promoting future doctors* increased the likelihood that they were motivated to train students in their own practice by almost twice as much as in the overall sample (OR: 34.8 vs. OR: 18.1). This could relate to the next generation of doctors or to the future doctors in their own practice.

With regard to incentives, there were no differences to the total sample, so that “knowledge-associated” incentives, e.g., independent, also medical didactic further training, access to literature and electronic databases could be offered.

Even if the CanMed roles explicitly specify the role of doctors as teachers, [26] – in the everyday life of GPs, patient care is in the priority. A frequently mentioned barrier of training students is the lack of time [10], [16], [27]. This was not explicitly addressed in our survey, but it is a fact that is already known or can be assumed by practising GPs in Thuringia. [28]. Accordingly, the non-teaching doctors more often agreed with the concerns that they *would not be able to treat the same number of patients* and that students *could disrupt practice operations.*

Benefits and challenges in connection with training students in one's own practice can and should be actively addressed in the recruitment and qualification of teaching physicians – such as the possibility of inspiring the next generation of physicians for one's own subject through teaching in the practice and, at the same time, keeping up with the current state of knowledge oneself. (Possible) barriers should also be addressed directly in order to develop constructive solutions. Here it is advisable to involve experienced teaching physicians who can help to deal with these challenges individually according to the “peer-to-peer” principle [8].

### 4.4. Strengths

Our survey had a high response rate and almost complete answers.

### 4.5. Limitations

Given the comparatively high response rate, it can be assumed that GPs interested in teaching were more likely to respond (response bias).

The group of “non-teaching physicians” is included in the total sample, therefore there is no strict group comparison.

## 5. Conclusion

The results describe a multi-faceted picture of GPs motivation to train students in their own practice. These can be used as a basis for attracting more teaching practices in Thuringia and beyond by addressing incentives – especially the possible gain in knowledge – and barriers. Further research on the success of the strategies used is needed.

## Competing interests

The authors declare that they have no competing interests. 

## Figures and Tables

**Table 1:**
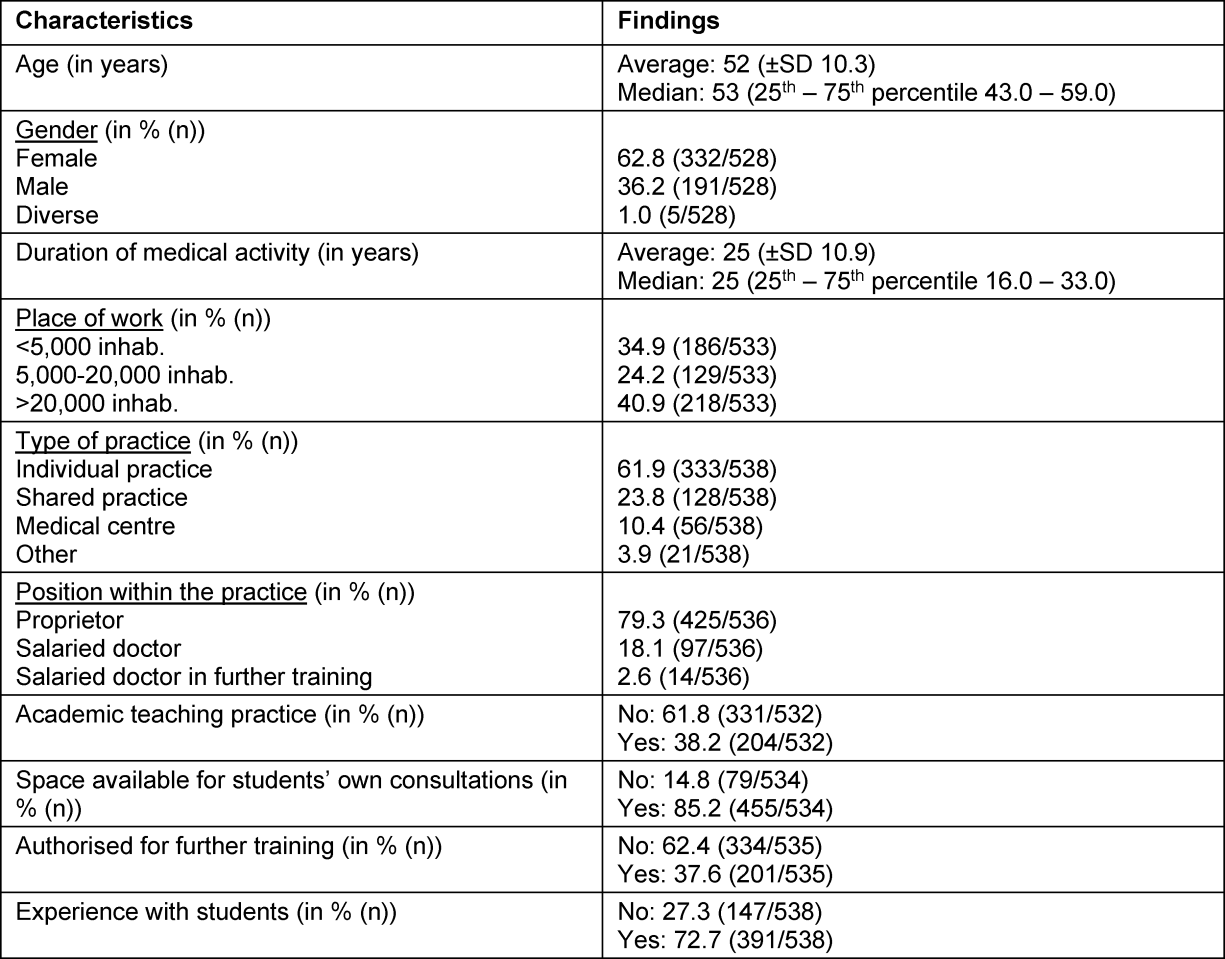
Selected socio-demographic/practice-related characteristics (n=538)

**Table 2:**
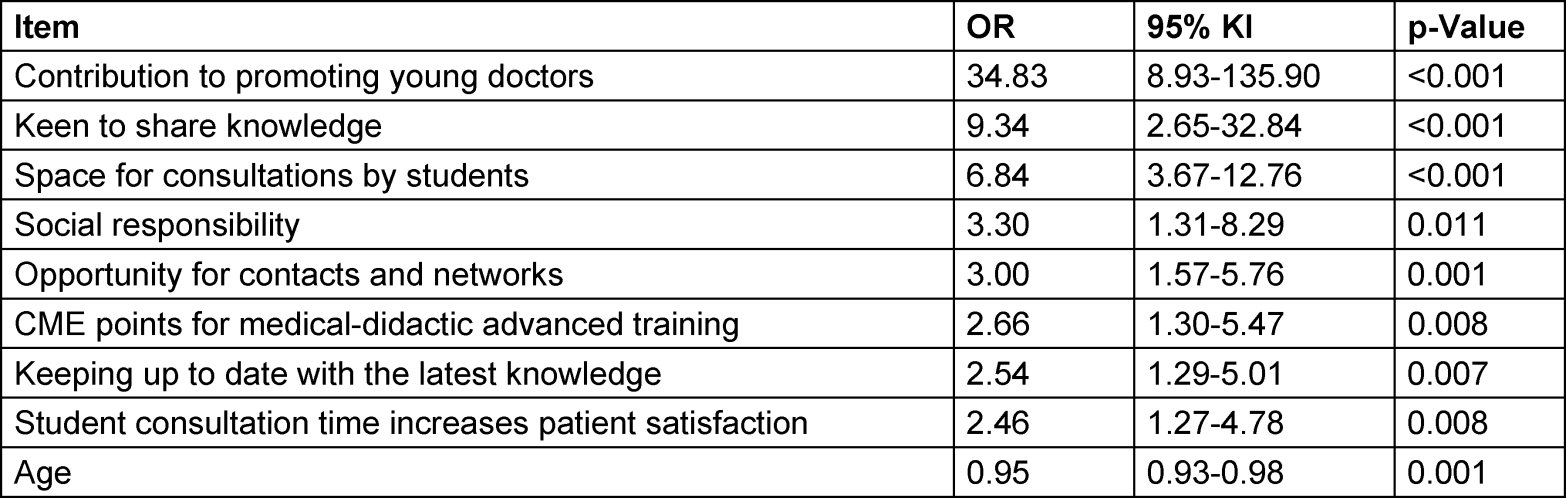
Significantly correlated factors influencing teaching motivation, subgroup of non-teaching physicians (n=331)
